# The expanding spectrum of modes of transmission of Zika virus: a global concern

**DOI:** 10.1186/s12941-016-0128-2

**Published:** 2016-03-03

**Authors:** Alfonso J. Rodriguez-Morales, Antonio Carlos Bandeira, Carlos Franco-Paredes

**Affiliations:** Public Health and Infection Research Group, Faculty of Health Sciences, Universidad Tecnologica de Pereira, Pereira, Risaralda Colombia; Organización Latinoamericana para el Fomento de la Investigación en Salud (OLFIS), Bucaramanga, Santander Colombia; Hospital Aliança and Faculdade de Tecnologia e Ciencias Medical School, Salvador, Brazil; Phoebe Putney Memorial Hospital, Albany, GA USA; Hospital Infantil de México, Federico Gómez, México, DF Mexico

During recent years, but particularly since 2015, concern on Zika virus has grown for multiple reasons, such as its association with the occurrence of Guillain–Barré syndrome and microcephaly [1, 2]. Nevertheless, in addition to all epidemiological implications of the outbreak in Latin America [3] the number of affected cases continue to rise and expected to reach over four million in 2016, adding to this the possibility of new modes of transmission.

**Fig. 1:**
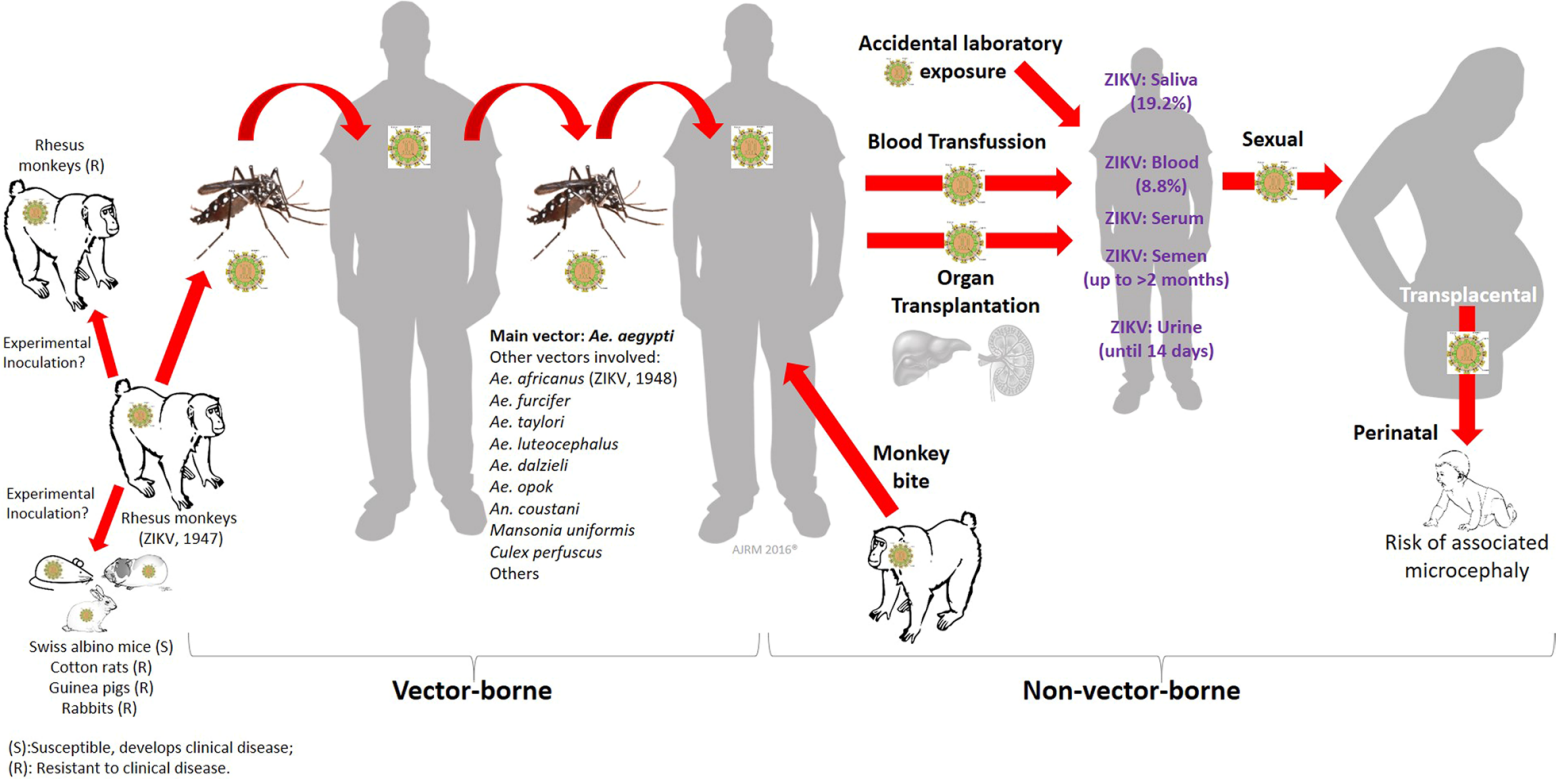
Summary of reported forms of transmission of Zika virus

Zika virus is a zoonotic pathogen, naturally and experimentally hosted in non-human primates [4] as well as experimentally in Swiss albino mices [5, 6]. Then, rhesus monkeys can be the source in natural habitats of human infections, through the bite of *Aedes aegypti* and *A. albopictus* (multiple other species and genus have been implicated) (Fig. 1), in infected monkeys and later transmission to susceptible human hosts.

Zika virus is predominantly a vector-borne disease (Fig. 1), although after the epidemics in the Pacific region it was clear that transplacental and perinatal transmission [7, 8] could also occur. However, the associated risk of microcephaly has been identified and highlighted much more recently as evidences continue to be added in different studies (Fig. 1) [8, 9].

Before the current epidemics not a single study raised that relationship, and, right now a significant number of pregnant women and their newborns are being monitored in Brazil [9], Colombia and other countries in the region for central nervous system anomalies. Zika has been detected in newborns, placenta and umbilical cords, as well in pregnant women by RT-PCR [8, 9]. There have been no reports detecting viable and potentially infective virus in breast milk up to now.

In addition to mother-to-child transmission, during the last decade, cases of sexual transmission have been reported [10–12], representing a non-vector borne form of transmission of Zika virus (Fig. 1). Zika virus has been detected in human saliva [13], blood, semen and urine [14]. It has been recently detected in semen and urine of, respectively, a patient after 62 days of infection [15] and another patient after 14 days of infection [16]. Also spread of the virus through blood transfusion and organ transplantation have been reported or suspected [17]. Zika virus infections have been documented through laboratory exposure [18].

Another emerging aspect of this zoonosis has been the possible transmission through bites of monkeys and other non-human primates (Fig. 1). This has also been recently reported [19].

Summarizing, Zika virus is primarily a vector-borne disease (mainly by *A. aegypti*), but there are also secondary modes of transmission (mother-to-child, sexual, blood transfusion, transplantation, non-human primate bites) (Fig. 1). This imply that prevention and control should consider all these ways of transmission, providing strategies to reduce new infections from this arbovirus that still need further basic, epidemiological and clinical assessment in order to clarify and understand its real impact on human health. Zika represents a real challenge for the medical and scientific community as well as for the world [20].
